# The Cooperative Induction of CCL4 in Human Monocytic Cells by TNF-α and Palmitate Requires MyD88 and Involves MAPK/NF-κB Signaling Pathways

**DOI:** 10.3390/ijms20184658

**Published:** 2019-09-19

**Authors:** Sardar Sindhu, Shihab Kochumon, Steve Shenouda, Ajit Wilson, Fahd Al-Mulla, Rasheed Ahmad

**Affiliations:** 1 Animal and Imaging Core Facility, Dasman Diabetes Institute, Dasman 15462, Kuwait; Sardar.Sindhu@dasmaninstitute.org; 2 Microbiolgy and Immunology, Dasman Diabetes Institute, Dasman 15462, Kuwait; shihab.kochumon@dasmaninstitute.org (S.K.); Ajit.Wilson@dasmaninstitute.org (A.W.); 3 Genetics & Bioinformatics, Dasman Diabetes Institute, Dasman 15462, Kuwait; fahd.almulla@dasmaninstitute.org

**Keywords:** CCL4, MIP-1β, TNF-α, palmitate, TLR4, MyD88, MAPK, NF-κB

## Abstract

Chronic low-grade inflammation, also known as metabolic inflammation, is a hallmark of obesity and parallels with the presence of elevated circulatory levels of free fatty acids and inflammatory cytokines/chemokines. CCL4/MIP-1β chemokine plays a key role in the adipose tissue monocyte recruitment. Increased circulatory levels of TNF-α, palmitate and CCL4 are co-expressed in obesity. We asked if the TNF-α/palmitate could interact cooperatively to augment the CCL4 production in human monocytic cells and macrophages. THP-1 cells/primary macrophages were co-treated with TNF-α/palmitate and CCL4 mRNA/protein expression was assessed using qRT-PCR/ELISA. TLR4 siRNA, a TLR4 receptor-blocking antibody, XBlue™-defMyD cells and pathway inhibitors were used to decipher the signaling mechanisms. We found that TNF-α/palmitate co-stimulation augmented the CCL4 expression in monocytic cells and macrophages compared to controls (*p* < 0.05). TLR4 suppression or neutralization abrogated the CCL4 expression in monocytic cells. Notably, CCL4 cooperative induction in monocytic cells was: (1) Markedly less in MyD88-deficient cells, (2) IRF3 independent, (3) clathrin dependent and (4) associated with the signaling mechanism involving ERK1/2, c-Jun, JNK and NF-κB. In conclusion, TNF-α/palmitate co-stimulation promotes the CCL4 expression in human monocytic cells through the mechanism involving a TLR4-MyD88 axis and MAPK/NF-κB pathways. These findings unravel a novel mechanism of the cooperative induction of CCL4 by TNF-α and palmitate which could be relevant to metabolic inflammation.

## 1. Introduction

Chronic low-grade inflammation, which is also called metabolic inflammation, plays a critical role in obesity-related insulin resistance and type-2 diabetes (T2D), as well as metabolic syndrome. Metabolic syndrome is regarded as a cluster of co-morbidities that enhance risks for cardiovascular disease, stroke and T2D. These co-morbidities may include hypertension, hyperglycemia, hypercholesterolemia, excessive abdominal obesity and increased triglyceride levels. In obesity, the white adipose tissue (WAT) is infiltrated by immune cells, especially by activated monocytes/macrophages which secrete proinflammatory cytokines and chemokines. Colonization of the adipose tissue by proinflammatory M1 macrophages is a striking feature of progressive obesity and involves the increased expression of tumor necrosis factor (TNF)-α, IL-1β, IL-6, IFN-γ, IL-8, IP-10, CCL2, CCL3, CCL4, CCL5, Eotaxins (CCL-11, 24, 26), CX3CL1 and several other chemokines. These inflammatory proteins may contribute, at various levels, to extravasation of circulatory monocytes into the adipose tissue and sustain metabolic inflammation which is pivotal to the development of insulin resistance. Macrophage inflammatory proteins (MIP) are the chemotactic cytokines that include MIP-1α (CCL3) and MIP-1β (CCL4). Both CCL3 and CCL4 orchestrate the immune responses to infection or inflammation [1]. During inflammation, these two chemokines can contribute to the expression of several other proinflammatory cytokines including TNF-α, IL-1β and IL-6 from activated macrophages and fibroblasts. While CCL3 expression and its regulation are well defined, relatively little is known about the induction and regulation of CCL4, especially during metabolic inflammation. CCL4 is a chemoattractant for critical immune regulatory cells such as monocytes/macrophages, T-lymphocytes, natural killer cells and dendritic cells [2,3]. CCL4 is produced by monocytes, T/B lymphocytes, neutrophils, fibroblasts and endothelial/epithelial cells [4,5]. CCL4 expression by neutrophils contributes to inflammation by recruiting other leukocytes at site(s) of inflammation which results in resolution of inflammation by macrophage-mediated efferocytosis or development of chronic inflammation. In addition to metabolic diseases, CCL4 is involved in the pathogenesis of systemic lupus erythematosus [6], multiple sclerosis [7], multiple myeloma [8], psoriasis [9], cystic fibrosis [10] and sarcoidosis [11]. Both immune and non-immune cells can express chemokines in response to TNF-α [12], IL-1β [13], leukotriene-B4 [14], complement fraction C5a [15], viruses [16], bacterial LPS [17,18] and free fatty acids [18,19].

Elevated CCL4 levels were reported in obesity and found to be correlated directly with metabolic inflammation [20]. However, mechanisms triggering the CCL4 levels in obesity are poorly understood. It is noteworthy that the increased circulatory levels of free fatty acids in obesity were reported to play a key role in chronic inflammation and insulin resistance [21,22]. The growing evidence now supports that free fatty acids can activate inflammatory signaling through different mechanisms including activation of the innate immune toll-like receptors (TLRs) [23,24]. TLR4, a member of the family of pattern-recognition receptors, activates the innate immune response after interaction with pathogen-associated molecular patterns including lipids, proteins, lipoproteins and LPS, as well as damage-associated signals or alarmins, e.g., high-mobility group box-1 protein (HMGB1) [25,26]. Binding of cognate ligands to TLRs activates two types of intracellular signaling pathways: One that requires myeloid differentiation factor-88 (MyD88) as an adaptor protein or another that involves the Toll/interleukin-1 receptor (TIR) domain-containing adaptor protein inducing IFNβ (TRIF). The MyD88-dependent signaling leads to downstream activation of nuclear factor kappa-B (NF-κB) and mitogen activated protein kinase (MAPK) pathways while the TRIF-dependent signaling leads to activation of interferon regulatory factor (IRF)-3 and its downstream signaling cascade [27].

The circulatory levels of free fatty acids are found to be high in obesity together with elevated inflammatory cytokines/chemokines and adipokines. Circulatory free fatty acids, such as palmitate, activate the TLR4-mediated signaling in immune cells and induce expression of proinflammatory cytokines including TNF-α, IL-6 and IL-1β [28]. Palmitate is known to induce the expression of chemokine CCL4 in human monocytic cells [29]. As well, increased levels of proinflammatory cytokine TNF-α have been found to correlate with insulin resistance [30]. Given that accumulating evidence points to the co-existence of increased circulatory levels of TNF-α, palmitate and CCL4 in obesity/T2D, we asked whether TNF-α and palmitate could cooperatively trigger the CCL4 production in human monocytic cells. Herein, we show that TNF-α and palmitate co-stimulation augments the CCL4 expression in THP-1 monocytic cells as well as primary human macrophages. We also report that this cooperative induction of CCL4 is TLR4-MyD88 dependent and involves NF-κB-MAPK-mediated signaling.

## 2. Results

### 2.1. TNF-α/Palmitate Cooperativity Triggers the CCL4 Expression in Human Monocytic Cells and Primary Human Macrophages

**Figure 1:**
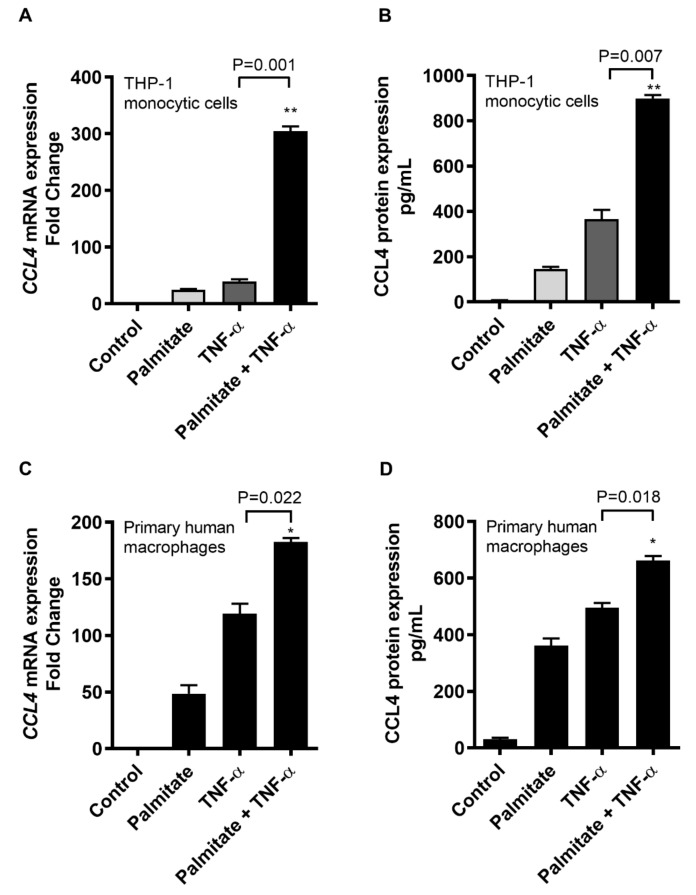
Palmitate and TNF-α cooperatively induce the CCL4 expression in human monocytic cells and primary macrophages. THP-1 monocytic cells and primary human macrophages were co-stimulated with palmitate and TNF-α following procedures as described in the materials and methods section, while each of the individual stimulations with vehicle, palmitate or TNF-α served as control. The data (shown as mean ± SEM) indicate that (**A**) CCL4 mRNA expression (*p* = 0.001) and (**B**) CCL4 secreted protein expression (*p* = 0.007) were highly promoted as THP-1 human monocytic cells were co-stimulated with TNF-α/palmitate compared to individual stimulations. Similarly, co-stimulation of primary human macrophages with TNF-α/palmitate also triggered the expression of (**C**) CCL4 mRNA (*p* = 0.022) and (**D**) CCL4 protein (*p* = 0.018) compared to individual stimulations. * Significant (*p* < 0.02), ** Very significant (*p* < 0.001).

We asked if the interaction between proinflammatory cytokine TNF-α and the saturated fatty acid palmitate could enhance the CCL4 expression in human monocytic cells. To this end, our data show that in THP-1 monocytic cells, CCL4 gene/protein expression was significantly promoted after co-treatment with TNF-α and palmitate as compared to treatment with TNF-α alone (CCL4 mRNA: *p* = 0.001; CCL4 protein: *p* = 0.007) (Figure 1A,B). To further see if this cooperativeness was reproducible in primary macrophages, we similarly treated the human macrophages and the data show that CCL4 mRNA and protein expressions were remarkably upregulated by TNF-α/palmitate co-stimulation as compared to TNF-α treatment alone (CCL4 mRNA: *p* = 0.022; CCL4 protein: *p* = 0.018) (Figure 1C,D).

### 2.2. CCL4 Co-Induction by TNF-α/Palmitate Requires TLR4 Signaling and Clathrin-Mediated Endocytosis

**Figure 2:**
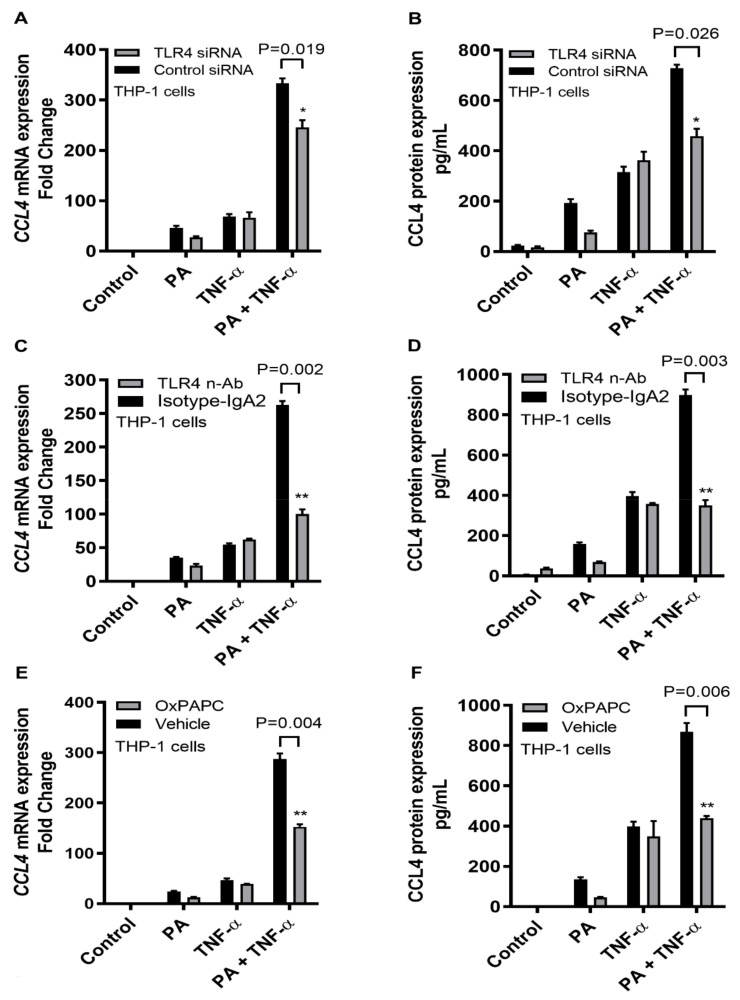
CCL4 co-induction by TNF-α/palmitate is TLR4 dependent. To investigate the involvement of TLR4 in the cooperative induction of CCL4 by TNF-α and palmitate, TLR4 signaling activity was blunted in THP-1 cells by TLR4 siRNA, the TLR4 neutralizing antibody or the TLR4 chemical inhibitor OxPAPC as described in the materials and methods section. The data (mean ± SEM) show that the cooperative effect of TNF-α/palmitate co-treatment was predominantly lost following siRNA-mediated ablation of TLR4 in THP-1 cells, resulting in significantly reduced expression of (**A**) CCL4 mRNA (*p* = 0.019) and (**B**) protein (*p* = 0.026) compared to control (scramble siRNA). Similarly, the cooperative induction of CCL4 by TNF-α/palmitate was abrogated after the TLR4 receptor was intercepted by treatment with an anti-TLR4 neutralizing antibody, displaying the diminished expression of (**C**) CCL4 mRNA (*p* = 0.002) and (**D**) CCL4 protein (*p* = 0.003) compared to the control (isotype-matched antibody). In addition, THP-1 cells treated with the the TLR4 inhibitor OxPAPC before TNF-α/palmitate co-stimulation also exhibited reduced expression of (**E**) CCL4 mRNA (*p* = 0.004) and (**F**) secreted CCL4 protein (*p* = 0.006) compared to the control (mock treatment). * Significant (*p* < 0.03), ** Very significant (*p* < 0.007).

Next, we demonstrate that the cooperative induction of CCL4 by TNF-α and palmitate involves the TLR4-dependent signaling and receptor-mediated endocytosis. To this effect, first TLR4 was ablated through genetic suppression by transfecting THP-1 cells with TLR4 siRNA and about 60% TLR4 suppression was achieved (Supplementary Figure S1). The data show that TNF-α/palmitate co-stimulation of TLR4-ablated monocytic cells resulted in significant abrogation of both CCL4 mRNA (*p* = 0.019) and protein (*p* = 0.026) (Figure 2A,B). Second, the TLR4 receptor on THP-1 cells was intercepted by incubating the cells with an anti-TLR4 neutralizing antibody before co-stimulation with TNF-α and palmitate, which also led to a significant reduction in CCL4 mRNA (*p* = 0.002) and protein (*p* = 0.003) compared to the isotype antibody-treated control (Figure 2C,D). Third, THP-1 cells were treated with the chemical inhibitor of TLR4 called OxPAPC prior to TNF-α/palmitate co-stimulation and again the CCL4 expression was downmodulated at both gene (*p* = 0.004) and protein (*p* = 0.006) levels (Figure 2E,F).

**Figure 3:**
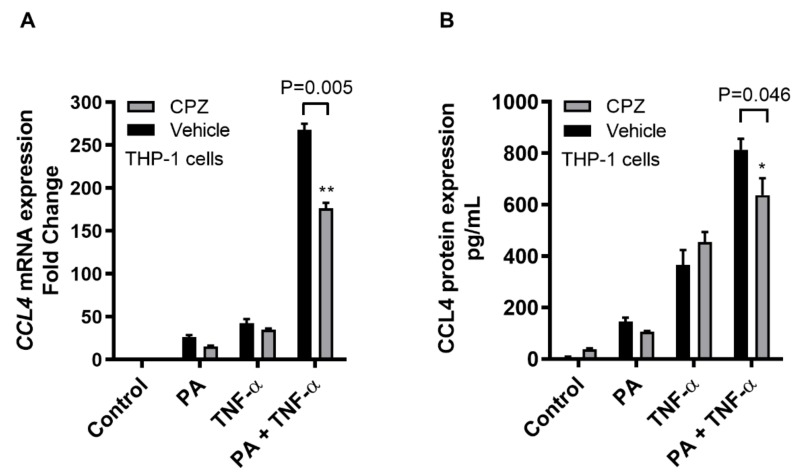
CCL4 co-induced by TNF-α/palmitate involves the clathrin-mediated endocytosis. To see if the CCL4 co-induction by TNF-α/palmitate involved the clathrin-mediated endocytosis, THP-1 monocytic cells were treated with specific inhibitor chlorpromazine (CPZ) before co-stimulation with TNF-α/palmitate as described in the materials and methods section. The data (mean ± SEM) show that CPZ-treated cells displayed significantly reduced expression of (**A**) CCL4 mRNA (*p* = 0.005) and (**B**) secreted CCL4 protein (*p* = 0.046) compared to the control (mock treatment). * Significant (*p* < 0.05), ** Very significant (*p* < 0.006).

To further see whether the CCL4 induction by TNF-α/palmitate co-treatment involved the clathrin-mediated endocytosis, receptor-mediated endocytosis was disrupted by pretreating THP-1 cells with a specific inhibitor chlorpromazine (CPZ). To this effect, our data show that CPZ treatment resulted in a significant suppression of CCL4 mRNA (*p* = 0.005) and secreted CCL4 protein (*p* = 0.046) (Figure 3), indicating that the clathrin-mediated endocytosis was involved in the cooperative induction of CCL4 in THP-1 monocytic cells by TNF-α/palmitate co-treatment. However, CPZ treatment may also affect the cell viability to a certain degree; therefore, the data on the effect of CPZ on cell viability using MTT assay are also shown (Supplementary Figure S2).

### 2.3. CCL4 Co-Induced by TNF-α/Palmitate is MyD88 Dependent

**Figure 4:**
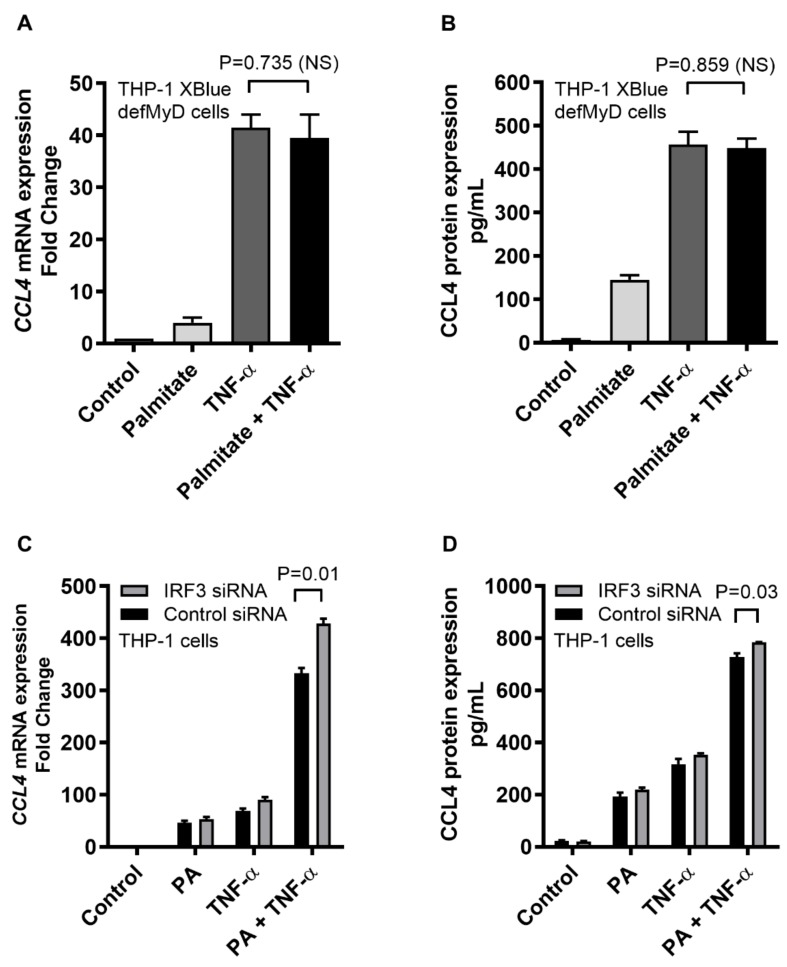
TNF-α/palmitate cooperativity for CCL4 induction is MyD88 dependent. To determine whether the MyD88 adaptor protein was required for the cooperative CCL4 expression, MyD88-deficient THP1-XBlue-defMyD cells were co-stimulated with TNF-α/palmitate. The data (mean ± SEM) show that the co-induction effect was lost in MyD88^−/−^ cells as TNF-α/palmitate co-stimulation induced similar expression of (**A**) CCL4 mRNA (*p* = 0.735) and (**B**) CCL4 secreted protein (*p* = 0.859) as compared to treatment with TNF-α alone. In parallel, IRF3 silencing was carried out as described in the materials and methods section and the data (mean ± SEM) show that IRF3 ablation did not downmodulate the expression of (**C**) CCL4 mRNA and (**D**) CCL4 secreted protein in IRF3-ablated cells compared to the control (scrambled siRNA-transfected cells). Together, these results support that the co-induction of CCL4 by TNF-α/palmitate in human monocytic cells is MyD88 dependent or IRF3 independent.

Following ligand binding, TLR4-downstream signaling requires the MyD88 (MyD88-dependent pathway) or the TRIF (IRF3-dependent/MyD88-independent pathway) as an adaptor protein. To determine the requirement for MyD88, MyD88-deficient THP1-XBlue™-defMyD cells were used. We found that the CCL4 expression in response to TNF-α/palmitate co-stimulation in MyD88-defective cells differed non-significantly at mRNA (*p* = 0.735) and protein (*p* = 0.859) levels compared to the CCL4 expression by TNF-α stimulation alone (Figure 4A,B). In parallel, almost similar induction of SEAP reporter-based NF-κB/AP-1 activity was observed after TNF-α/palmitate co-stimulation as compared to TNF-α stimulation alone (*p* = 0.06) (Supplementary Figure S3). To assess the involvement of IRF3 pathway, IRF3 siRNA was transfected in THP-1 monocytic cells and about 56% genetic suppression of IRF3 was achieved (Supplementary Figure S4). Next, stimulation of the IRF3-ablated cells with TNF-α/palmitate co-treatment did not result in a reduced CCL4 expression as compared to control (scrambled siRNA). Rather, CCL4 expression in the IRF3-ablated cells was higher at both mRNA (*p* = 0.01) and protein (*p* = 0.03) levels as compared to the control (Figure 4C,D). Altogether, these data indicate that the cooperative induction of CCL4 by TNF-α/palmitate co-stimulation in THP-1 monocytic cells is MyD88 dependent and does not require IRF3 as an adaptor protein for TLR4-downstream signaling.

### 2.4. CCL4 Expression by TNF-α/Palmitate Co-Stimulation Involves the MAPK/NF-κB Signaling Pathways

**Figure 5:**
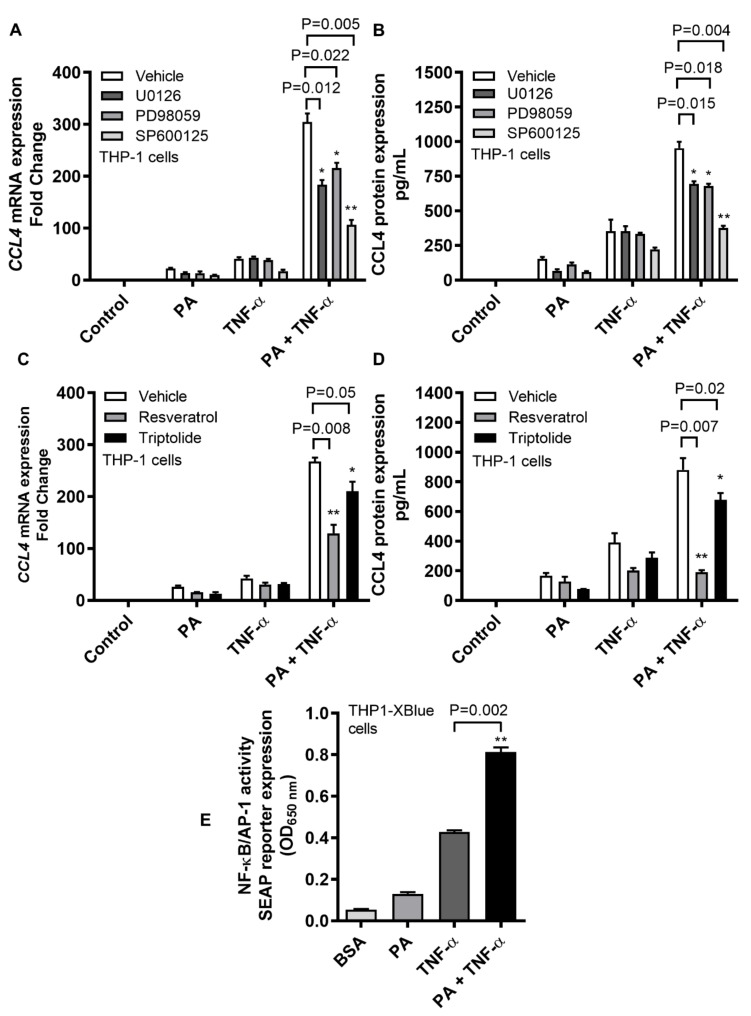
CCL4 co-induction by TNF-α/palmitate involves the MAPK/NF-κB signaling pathways. To see whether the TLR4 downstream signaling involved MAPK/NF-κB pathways, THP-1 cells were pre-treated with specific pathway inhibitors before co-stimulation with TNF-α/palmitate as described in the materials and methods section. (**A**) The data (mean ± SEM) show that following TNF-α/palmitate co-stimulation, CCL4 mRNA expression was significantly suppressed in cells that were treated with MAPK inhibitors including U0126 (*p* = 0.012), PD98059 (*p* = 0.022) and SP600125 (*p* = 0.005) compared to the control (mock). (**B**) As expected, CCL4 protein expression following TNF-α/palmitate co-simulation was also abrogated in cells that were treated with U0126 (*p* = 0.015), PD98059 (*p* = 0.018) and SP600125 (*p* = 0.004) as compared to the control. Likewise, NF-κB-mediated signaling was intercepted by using specific pathway inhibitors including resveratrol and triptolide. (**C**) The data (mean ± SEM) indicate that CCL4 mRNA expression was significantly downmodulated in THP-1 monocytic cells that were treated with resveratrol (*p* = 0.008) and triptolide (*p* = 0.05) compared to the control (mock). (**D**) Similarly, CCL4 secreted protein was also reduced in cells that were treated with resveratrol (*p* = 0.007) and triptolide (*p* = 0.02) compared to control. (**E**) To further confirm that TNF-α/palmitate co-stimulation induced the NF-κB/AP-1 activity, THP1-XBlue cells expressing the NF-κB/AP-1 promoter linked to the SEAP reporter were used. The data (mean ± SEM) show that co-stimulation with TNF-α/palmitate induced a stronger NF-κB/AP-1 activity as compared to stimulation with TNF-α alone (*p* = 0.002). * Significant (*p* < 0.05), ** Very significant (*p* < 0.009).

TLR4 downstream signaling may lead to activation of the MAPK and NF-κB pathways. We next investigated if the cooperative induction of CCL4 by TNF-α/palmitate co-stimulation involved signaling through the MAPK and/or NF-κB pathways. To this end, our data show that following TNF-α/palmitate co-stimulation, both the gene and protein expression of CCL4 were significantly abrogated in THP-1 cells that were treated with MAPK pathway inhibitors such as U0126 (mRNA: *p* = 0.012; protein: *p* = 0.015), PD98059 (mRNA: *p* = 0.022; protein: *p* = 0.018) and SP600125 (mRNA: *p* = 0.005; protein: *p* = 0.004) compared to the mock (Figure 5A,B). Likewise, CCL4 mRNA and protein expressions were significantly downmodulated following TNF-α/palmitate co-stimulation in cells that were treated with NF-κB pathway inhibitors including resveratrol (mRNA: *p* = 0.008; protein: *p* = 0.007) and triptolide (mRNA: *p* = 0.05; protein: *p* = 0.02) as compared to the control (Figure 5C,D). In addition, we also used THP1-XBlue cells that were stably transfected with the secreted embryonic alkaline phosphatase (SEAP) reporter construct linked to NF-κB/AP-1 promoter activity and, in this case, SEAP reporter activity was significantly upregulated when cells were co-stimulated with TNF-α/palmitate as compared to stimulation with TNF-α alone (Figure 5E) (*p* = 0.002).

**Figure 6:**
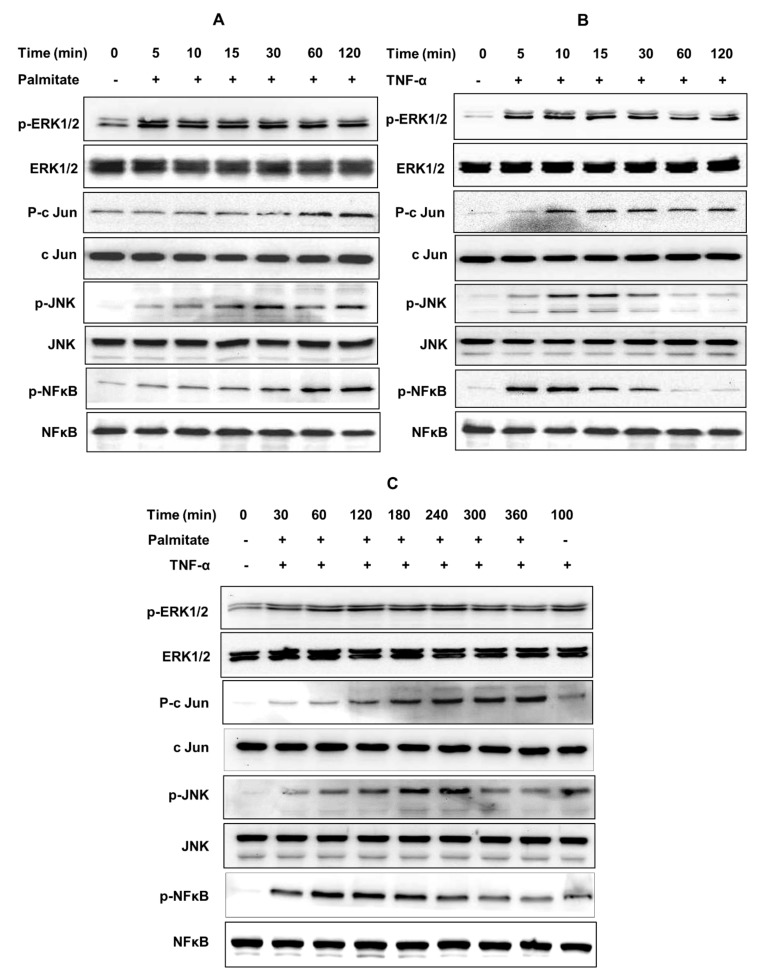
TNF-α/palmitate cooperativeness for CCL4 expression implicates ERK-1/2, c-Jun, JNK and NF-κB phosphorylation. To further verify the involvement of MAPK/NF-κB signaling pathways, phosphorylation of the transcription factors including ERK-1/2, c-Jun, JNK and NF-κB was assessed by western blotting as described in the materials and methods section. The blots show the extended phosphorylation timings for ERK1/2, c-Jun, JNK and NF-κB signaling proteins in cells that were co-stimulated with TNF-α/palmitate as compared to timings of respective phosphorylated proteins in cells that were treated with either palmitate or TNF-α. (**A**) Palmitate treatment induced phosphorylation of ERK1/2, c-Jun, JNK and NF-κB for up to 120 min each. (**B**) TNF-α treatment induced phosphorylation of ERK1/2 and c-Jun, each for up to 120 min, phosphorylation of JNK for up to 15 min and phosphorylation of NF-κB for up to 10 min. (**C**) TNF-α/palmitate co-stimulation induced phosphorylation of these signaling proteins for extended time periods, i.e., phosphorylation of both ERK1/2 and c-Jun for up to 360 min, phosphorylation of JNK for up to 240 min and phosphorylation of NF-κB for up to 180 min. Note: Phospho- and total- ERK1/2: 42, 44 KDa; Phospho- and total- c-Jun: 48 KDa; Phospho- and total- JNK: 46, 54 KDa; and Phospho- and total- NF-κB: 65 KDa.

To further confirm whether the MAPK/NF-κB signaling pathways were involved in the cooperative induction of CCL4 by TNF-α and palmitate, phosphorylation of transcription factors including extracellular signal-regulated kinase (ERK)-1/2, c-Jun, c-Jun N-terminal kinase (JNK) and NF-κB was assessed by western blotting. To this effect, as evident from blots, palmitate-induced phosphorylation of ERK1/2, c-Jun, JNK and NF-κB was sustained up to 120 min each (Figure 6A). TNF-α induced phosphorylation of ERK1/2 and c-Jun for up to 120 min, phosphorylation of JNK until 15 min and NF-κB phosphorylation until 10 min (Figure 6B). However, phosphorylation of these signaling proteins was sustained for longer time periods when cells were co-stimulated with TNF-α/palmitate. In this case, phosphorylation of ERK1/2 and c-Jun was prolonged up to 360 min, phosphorylation of JNK up to 240 min and phosphorylation of NF-κB was extended up to 180 min (Figure 6C). The normalization data regarding ratios of phosphorylated to respective total proteins are not shown. The graphics of bands’ densitometric data are shown regarding the MAPK/NF-κB phosphorylation levels following treatments with palmitate (Supplementary Figure S5), TNF-α (Supplementary Figure S6) and palmitate/TNF-α combined (Supplementary Figure S7).

## 3. Discussion

In this study we show, for the first time to our knowledge, that the cooperative interaction between an inflammatory cytokine (TNF-α) and a saturated fatty acid (palmitate) triggers CCL4 expression in the human monocytic cells. In obesity/T2D, visceral WAT undergoes the expansion that involves adipocyte hypertrophy/hyperplasia and neovascularization with drastic changes in the number and function of immune cells including monocytes/macrophages, T/B-lymphocytes, neutrophils, mast cells, eosinophils, regulatory T cells, NK cells and iNKT cells, especially with an anti-inflammatory (M2) to inflammatory (M1) macrophage shift in the adipose tissue [31]. It is believed that obesity-induced changes in the adipose tissue expression of cytokines/chemokines play a key role to offset the balance of macrophage polarization from M2-favoring alternative activation to M1-favoring classical activation. Another remarkable change that occurs in obesity is the increase in the circulating levels of free fatty acids [22]. Notably, the elevated circulatory levels of TNF-α, palmitate and CCL4 remain an important concern with regard to metabolic inflammation; however, it is unclear whether simultaneous exposure to TNF-α and palmitate can promote CCL4 expression in monocytic cells. CCL4 directly contributes to the obesity-associated metabolic inflammation by influencing the migration and recruitment of monocytes into the adipose tissue [32]. TNF-α has also been reported as a major player in metabolic inflammation and insulin resistance [33]. We have previously shown that palmitate induces CCL4 expression in human monocytic cells [29]. Herein, we further show that the cooperative interaction between TNF-α and palmitate triggers CCL4 expression in human monocytic cells as well as primary human macrophages. To our knowledge, no other study so far has elucidated the cooperative interaction between TNF-α and palmitate for triggering the CCL4 expression in human monocytic cells. Given that both TNF-α and CCL4 are critical players in metabolic inflammation, the present study contributes important novel data by showing how TNF-α/palmitate co-stimulation augments CCL4 production in monocytic cells/macrophages. These data are consistent, at least in part, with the previous study reporting enhanced CCL2 expression via a synergy between TNF-α and palmitate through the mechanism involving TRIF/IRF3-dependent signaling [34].

The mounting evidence now supports that TLRs’ expression is modulated positively by obesity/T2D [35,36,37,38,39]. Specifically, TLR4 has been recognized as a nutrient sensor and the main receptor that is activated by free fatty acids and endotoxin (LPS) [23,25]. Therefore, we sought to determine whether TNF-α and palmitate could cooperatively augment the CCL4 expression in monocytic cells via TLR4-mediated signaling. In this regard, first we show that siRNA-mediated genetic suppression of TLR4 in THP-1 cells led to a significant abrogation of CCL4 mRNA and protein expression. Second, masking the TLR4 receptor by labeling cells with an anti-TLR4 neutralizing antibody also downmodulated the CCL4 mRNA and protein expression. Third, treating THP-1 monocytic cells with TLR2/4 inhibitor OxPAPC also suppressed the expression of CCL4 mRNA and secreted protein. Taken together, multiple experimental approaches used in our study congruently show that TLR4 is essentially required for synergic induction of CCL4 in monocytic cells by co-treatment with TNF-α and palmitate. Clathrin-mediated endocytosis is a key process that regulates the vesicular trafficking or transport of a wide range of cargo molecules from the cell surface to the interior. To this effect, we found that the cooperative induction of CCL4 by TNF-α/palmitate was significantly abrogated after cells were pre-treated with chlorpromazine, which is a clathrin pathway inhibitor. These findings are in agreement with a previous report showing that the clathrin-dependent endocytosis was critical for the TLR3- and TLR4-mediated Toll-IL-1R domain-containing adaptor molecule-1 (TICAM-1) signaling [40]. However, the induction of TLR4 translocation may involve clathrin or other mechanisms, e.g., CD14 and phosphatidylinositol metabolism. Together, our results indicate that the cooperative induction of CCL4 by TNF-α/palmitate co-stimulation involves TLR4-mediated signaling and clathrin-mediated endocytosis.

The binding of a cognate ligand to TLR4 may induce downstream signaling through the MyD88-dependent or -independent (IRF3-dependent) pathways [41]. We next investigated whether the cooperative CCL4 production by TNF-α/palmitate co-stimulation was MyD88 dependent or independent (i.e., IRF3 dependent). In this regard, we found that: (1) The cooperative effect of CCL4 induction was lost in MyD88-defecient THP1-XBlue defMyD cells; and (2) IRF3 genetic suppression in THP-1 cells by siRNA did not abrogate the CCL4 triggering effect of TNF-α/palmitate co-stimulation. Collectively, these data show that the cooperative induction of CCL4 was MyD88 dependent and did not require the IRF3 adaptor protein for TLR4-downstream signaling. On the contrary, we previously found that another CC-motif chemokine CCL2 was cooperatively enhanced in monocytic cells by co-stimulation with TNF-α and palmitate through the TRIF/IRF3-dependent (or MyD88-independent) mechanism [34]. Overall, it may be inferred that TNF-α/palmitate co-stimulation may induce various CC-motif chemokines in monocytic cells, involving either the MyD88-dependent or -independent mechanism.

Following TLR activation and MyD88 or TRIF/IRF3 recruitment, the initiation of signal transduction through MAPKs and/or NF-κB pathways may lead to the expression of cytokines and chemokines. MAPKs/NF-κB are the classical signaling cascades that are induced downstream of TLR4 activation. The data obtained from our experiments involving pathways’ inhibitors confirm that the cooperative induction of CCL4 by TNF-α/palmitate co-stimulation was dependent on MAPK-/NF-κB-mediated signaling. To this end, the induction of NF-κB/AP-1 activity was further confirmed by enhanced SEAP reporter expression in THP1-XBlue cells. Consistent with our findings, TLR4 and TNF-αR agonists were found to activate the NF-κB/AP-1 signaling pathways in monocytic cells [42,43]. We further show that the TNF-α/palmitate co-stimulation in monocytic cells induced a relatively prolonged phosphorylation of transcription factors including ERK1/2, c-Jun, SAPK/JNK and NF-κB as compared to stimulations with palmitate or TNF-α alone, which may explain how the cooperativeness between palmitate and TNF-α augments the CCL4 expression in these cells. This also indicates that a saturated fatty acid such as palmitate, like an endotoxin (LPS), could engage with the TLR4 nutrient sensor and initiate signals through NF-κB, c-Jun, SAPK/JNK, ERK1/2 and MAPKs. All of these transcription factors have been reported to play key roles in metabolic inflammation and insulin resistance [29,44,45]. Similarly, LPS-induced cytokine/chemokine gene expression has been shown to activate the TLR4-/NF-κB-mediated signaling [46]. In relation to CCL4 expression in various cell types, NF-κB-mediated CCL4 expression has been reported in the human monocytic cells [29,47], chondrocytes [48] and neuronal cells [49].

In summary, our data show that the cooperative interaction between TNF-α and palmitate leads to an excessive production of the chemokine CCL4 in human monocytic cells and primary human macrophages. This CCL4 induction in monocytic cells was found to be TLR4 and MyD88 dependent and involved the MAPK-/NF-κB-mediated signaling. These findings support a plausible CCL4 amplification mechanism that may contribute to metabolic inflammation.

## 4. Materials and Methods

### 4.1. Cell Cultures and Stimulation

Human THP-1 monocytic cell line was obtained from American Type Culture Collection (ATCC, Manassas, VA, USA) and cells were cultured in RPMI-1640 complete medium (Gibco, Life Technologies, Grand Island, NY, USA) containing 10% FBS (Gibco, Life Technologies), 2 mM glutamine (Gibco, Invitrogen, Grand Island, NY, USA), 1 mM sodium pyruvate, 10 mM HEPES, 50 U/mL penicillin, 50 μg/mL streptomycin and 100 μg/mL Normocin, (Gibco, Invitrogen), and cells were incubated at 37 °C with humidity and 5% CO_2_. THP1-XBlue cells stably expressing NF-κB/AP-1 inducible and SEAP reporter, as well as THP 1 cells deficient in MyD88 activity, known as THP1-XBlue™-defMyD cells or MyD88^-/-^ THP-1 cells, were purchased from a commercial source (InvivoGen, San Diego, CA, USA). THP1-XBlue cells were cultured in RPMI-1640 complete medium containing zeocin (200 μg/mL; InvivoGen) to maintain stable expression of the NF-κB/AP-1 driven SEAP reporter. THP1-XBlue™-defMyD cells were cultured in RPMI-1640 complete medium containing Zeocin (200 μg/mL) and HygroGold (100 μg/mL; InvivoGen). Before stimulating, THP-1 cells were transferred to normal medium and plated in 12-well plates (Costar, Corning Incorporated, Oneonta, NY, USA) at a cell density of 1 × 10^6^ cells per well unless otherwise stated. Cells were stimulated with palmitate (200 μM; Sigma, San Diego, CA, USA) and/or TNF-α (10ng/mL; Sigma) or 0.1% BSA (Sigma) and incubated at 37 °C for 24 h. Cells were harvested for RNA isolation and culture supernatants for secreted CCL4 measurement.

### 4.2. Isolation of Peripheral Blood Mononuclear Cells (PBMCs), Primary Macrophage Differentiation and Stimulation

Human peripheral blood samples (40 mL each) were collected by venipuncture using EDTA vacutainer tubes from healthy donors at the Dasman Diabetes Institute (DDI) phlebotomy unit and following written informed consent of the participants and study approval by the DDI ethics committee. PBMCs were isolated using the HistoPaque density gradient method as described [50]. PBMCs were seeded in 6-well plates (Costar, Corning Incorporated) at a cell density of 3 × 10^6^ cells per well and cultured in a starvation (serum-free) medium at 37 °C for 3 h. Non-adherent cells were removed by gentle repeated washing with a serum-free culture medium and adherent macrophages were further incubated for 24 h in RPMI medium containing 2% FBS. Cells were then stimulated with palmitate (200 μM; Sigma) and/or TNF-α (10 ng/mL; Sigma) or 0.1% BSA and incubated at 37 °C for 24 h. Later, cells were harvested for total RNA isolation and culture media were collected for secreted CCL4 measurement.

### 4.3. TLR4 Neutralization or Chemical Inhibition

THP-1 monocytic cells were labeled by treating with neutralizing anti-TLR4 IgG1 mAb (2 μg/mL; Cat. #mab2-htlr4, InvivoGen) or isotype-matched control IgG1 mAb (2 μg/mL; Cat. #mabg1-ctrlm, InvivoGen) for 40 min. Antibody-labeled cells were stimulated with palmitate or 0.1% BSA as described before and incubated at 37 °C for 24 h. Both cells and culture media were collected. OxPAPC is known to inhibit the signaling via TLR2/TLR4. It competes with the accessory proteins such as CD14, LBP and MD2 and inhibits the TLR2/4-mediated signaling. To assess the role of TLR4, THP-1 monocytic cells were treated in triplicate wells of 12-well plates (10^6^ cells/mL/well) with palmitate (200 μM; Sigma, CA, USA) and/or TNF-α (10 ng/mL; Sigma), with or without OxPAPC (30 μg/mL; Cat. # tlrl-oxp1, InvivoGen), and incubated at 37 °C for 24 h. Cells were harvested for total RNA extraction and supernatants were collected for measuring the secreted CCL3 protein.

### 4.4. Trafficking Inhibition

To study the internalization mechanism, THP-1 monocytic cells seeded in triplicate wells of 12-well plates (10^6^ cells/mL/well) were pre-incubated at 37 °C for 1 h with 20 μM CPZ, a trafficking inhibitor of clathrin-mediated endocytosis and the vehicle-treated cells served as control. Later, cells were treated with palmitate (200 μM; Sigma) and/or TNF-α (10 ng/mL; Sigma) or 0.1% BSA (mock) and incubated at 37 °C for 24 h to allow endocytosis. Cells were harvested for total RNA extraction and supernatants were collected for measuring the secreted CCL3 protein.

### 4.5. Measurement of Cell Viability by MTT Assay

MTT assays were performed using the TACS MTT ^®^ Cell Proliferation Assay Kit (Cat#: 4890-25-K, Trevigen, city, state, USA) and following the manufacturer’s instructions. Briefly, 10^6^ cells in 100 µL serum-free medium were dispensed in 96-well plates and incubated at 37 °C for 18 h, with or without CPZ. Later, 10 µL of MTT reagent was added to each well and incubated for 4 h until the purple dye was clearly visible. Then, 100 µL of detergent reagent (Cat#: 4890-25-02) was added to each well and incubated again at 37 °C for 4 h. The absorbance was read at 570 nm wavelength and the percentage of viable cells for each treatment was calculated as compared to viable cells in untreated control.

### 4.6. Measurement of NF-κB/AP-1 Activity

THP1-XBlue cells are THP-1 monocytic cells that are stably transfected with a reporter construct expressing the SEAP gene under the control of a promoter induced by transcription factors including NF-κB and AP-1. Thus, the cell stimulation results in the NF-κB and AP-1 activation and SEAP expression. THP1-XBlue cells were stimulated with palmitate (200 μM) and/or TNF-α (10 ng/mL) or 0.1% BSA (mock) and cell cultures were incubated at 37 °C for 24 h. The SEAP levels were measured using ELISA by incubating the culture supernatants for 3 h with QUANTI-Blue™ solution (InvivoGen) and measuring absorption at 650 nm wavelength.

### 4.7. siRNA-Mediated Genetic Suppression

For siRNA-mediated TLR4 or IRF3 genetic suppression, THP-1 cells were washed, resuspended in 100 μL nucleofector solution (Amaxa Nucleofector™ Kit V, Lonza Bioscience, Cologne, Germany) and transfected separately with TLR4 siRNA or IRF3 siRNA (30 nM each; OriGene Technologies Inc. MD, USA), scrambled (control) siRNA (30 nM; OriGene Technologies Inc. MD, USA) and pmaxGFP (0.5 μg; Amaxa Nucleofector™ Kit V, Lonza Bioscience, Cologne, Germany). All transfections were performed using Amaxa Nucleofector Kit V reagents for THP-1 cell line (Lonza Bioscience, Cologne, Germany) and Amaxa Electroporation System (Amaxa Inc., Cologne, Germany) following the manufacturer’s instructions. At 36 h post-transfection, cells were treated with palmitate (200 μM) and/or TNF-α (10 ng/mL) or 0.1% BSA and incubated at 37 °C for 24 h. Cells were harvested for RNA isolation and culture supernatants were collected for CCL4 protein measurement. The transfection efficiency of siRNA was determined by fluorescence microscopy and the genetic suppression of TLR4 or IRF3 was assessed by real-time PCR using TLR4 or IRF3 gene-specific primer probes.

### 4.8. Real-Time RT-PCR

Total cellular RNA was extracted by using RNeasy Mini Kit (Qiagen, Valencia, CA, USA) and following the manufacturer’s instructions. Then, complementary DNA (cDNA) was synthesized by using 1 μg of total RNA and following guidelines from the high-capacity cDNA reverse transcription kit (Applied Biosystems, Foster city, CA, USA). For carrying out each real-time PCR, 50 ng of cDNA template was used and amplified with Inventoried TaqMan Gene Expression Assay products (CCL4/MIP-1 β: Hs99999148_m1; GAPDH: Hs03929097_g1; TLR-4: Hs00152939_mL) comprised of two gene-specific primers, a TaqMan MGB probe (6-FAM dye-labeled) and TaqMan^®^ Gene Expression Master Mix (Applied Biosystems) using 7500 Fast Real-Time PCR System (Applied Biosystems, CA, USA). Target mRNA levels were normalized against housekeeping GAPDH mRNA and the CCL4 mRNA expression relative to control was calculated by using the 2^−ΔΔCT^ method. The relative mRNA expression was expressed as fold expression over the average control gene expression. The expression level in control treatment was assumed as 1.

### 4.9. ELISA

The CCL4 secreted protein levels were measured in supernatants of palmitate- and/or TNFα-stimulated THP-1 monocytic cells by using sandwich ELISA and following the manufacturer′s instructions (R&D systems, Minneapolis, MN, USA).

### 4.10. Western Blotting

THP-1 monocytic cells were treated with palmitate (200 μM) and/or TNF-α (10 ng/mL) for the incubation times as indicated, harvested and lysed by incubation with lysis buffer (10× Lysis Buffer, Cell signaling Technology Inc., Danvers, Massachusetts, MA, USA) for 30 min. The lysates were centrifuged at 14000 × g for 10 min and supernatants were collected. Protein concentration was measured in lysates by using QuickStart Bradford Dye Reagent, 1× Protein Assay kit (Bio-Rad Laboratories Inc., Hercules, CA, USA). Protein samples (20 μg each) were mixed with loading buffer, heated at 95 °C for 5 min and resolved by 12% sodium dodecyl sulfate polyacrylamide gel electrophoresis (SDS-PAGE). Proteins were transferred by electro blotting to an Immuno-Blot PVDF membrane (Bio-Rad Laboratories). The membranes were blocked by treating with 5% non-fat milk in PBS for 1 h, followed by overnight incubation at 4 °C with primary antibodies (1:1000 diluted) against p-ERK1/2, p-c-Jun, p-SAPK/JNK, p-NF-κB, ERK1/2, c-Jun, SAPK/JNK and NF-κB (Cell Signaling Technology Inc., MA, USA). Blots were washed thrice by using Tris buffered saline with 0.1% TBST and incubated for 2 h with an HRP-conjugated secondary antibody (Promega, WI, USA). Immunoreactive bands were developed by using Amersham ECL Plus Western Blotting Detection System (GE Health Care, Buckinghamshire, UK) and visualized by Molecular Imager^®^ VersaDoc™ MP Imaging Systems (Bio-Rad Laboratories).

### 4.11. Statistical Analysis

Data are shown as mean ± SEM values and statistical analysis was performed by using GraphPad Prism (Version 6.05, San Diego, CA, USA). Group variances were compared by using one-way ANOVA, while unpaired the Student t-test was used to compare the group means. All *p*-values <0.05 were considered as significant.

## Supplementary Materials

Supplementary materials can be found at https://www.mdpi.com/1422-0067/20/18/4658/s1.
