# Supplementary material for: The Cooperative Induction of CCL4 in Human Monocytic Cells by TNF-α and Palmitate Requires MyD88 and Involves MAPK/NF-κB Signaling Pathways
Source: Int J Mol Sci. 2019 Sep 19;20(18):4658. doi: 10.3390/ijms20184658 (PMC6770648; doi:10.3390/ijms20184658)
Supplement: Supplementary file 1 [file ijms-20-04658-s001.pdf]

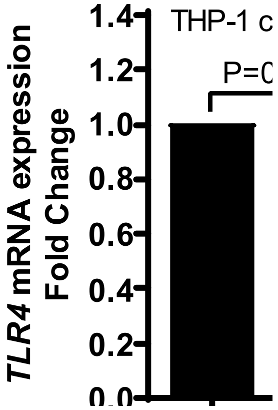

Figure S1. siRNA-mediated TLR4 genetic suppression in THP-1 cells

TLR4 genetic ablation was achieved by transfecting THP-1 human monocytic cells with TLR4-specific siRNA while the cells transfected with scrambled siRNA served as control. *TLR4* mRNA expression was determined using real-time RT-PCR as described in materials and methods. The data (mean±SEM) show significant target gene suppression in TLR4 siRNA transfected cells compared to scrambled control (P=0.001).

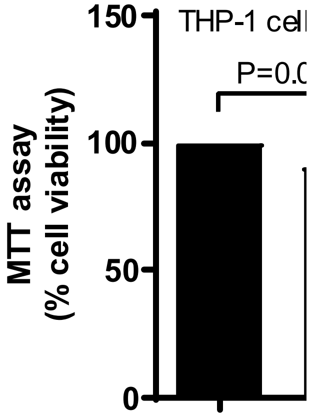

FigureS2. Cell viability by MTT assay

MTT assays were performed using the TACS MTT ® Cell Proliferation Assay Kit and following the recommended protocol as described in materials and methods. The data (mean±SEM) show % cell viability difference between chlorpromazine (CPZ) treated and untreated cells (P=0.04).

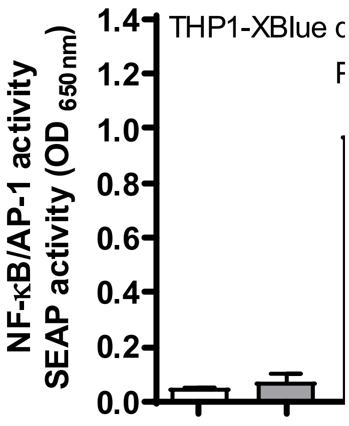

Figure S3. NF-kB/AP-1 activity in MyD88-deficient cells

MyD88-deficient THP-1 cells (THP1-XBlue™-defMyD cells) expressing NF-κB/AP-1 inducible secreted embryonic alkaline phosphatase (SEAP) reporter (OD measured at 650nm) were stimulated with palmitate, TNF-α, TNF-α/palmitate combined or BSA and incubated at 37°C for 24h as described in materials and methods. The data (mean±SEM) show that TNF-α/palmitate co-stimulation of $MyD88^{-}/^{-}$ THP-1 cells did not induce a higher NF-κB/AP-1 activity compared to stimulation by TNF-α alone.

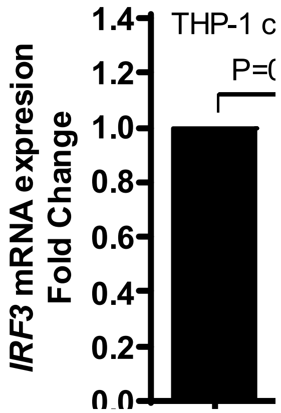

Figure S4. siRNA-mediated IRF3 genetic ablation in THP-1 cells

IRF3 genetic suppression was achieved by transfecting THP-1 human monocytic cells with IRF3-specific siRNA while the cells transfected with scrambled siRNA served as control. *IRF3* mRNA expression was determined using real-time RT-PCR as described in materials and methods. The data (mean±SEM) show significant target gene suppression in IRF3 siRNA transfected cells compared to scrambled control (P=0.002).

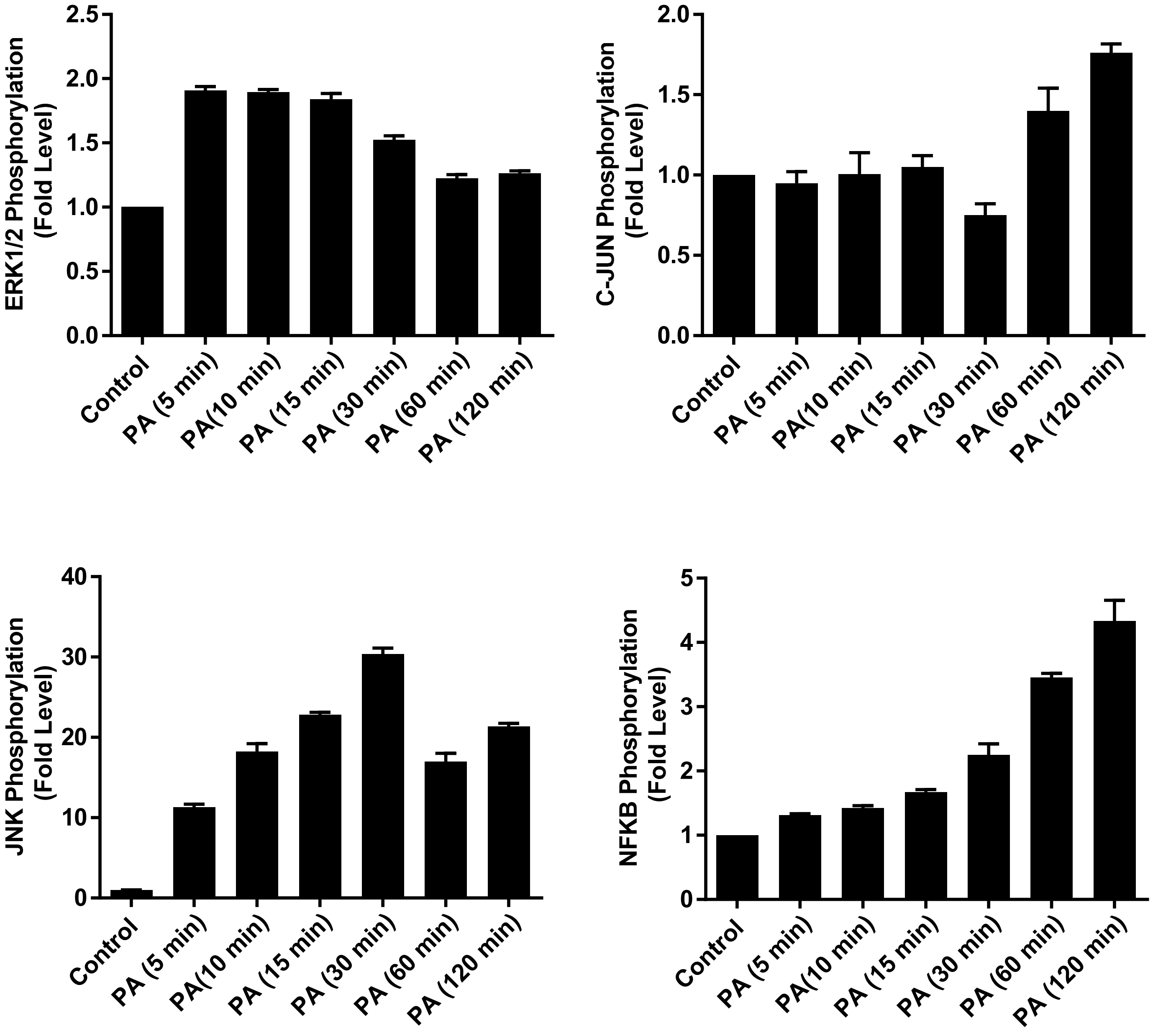

Figure S5. MAPK/NF-κB phosphorylation levels following palmitate treatment

THP-1 monocytic cells were treated with palmitate (200µM) for 5, 10, 15, 30, 60, and 120 min. Cell lysates were centrifuged, and supernatants were collected for protein measurement. Samples (20µg each) were loaded and resolved by SDS-PAGE (12%) as described in materials and methods. Electroblotted proteins were developed to measure the expression of phosphorylated ERK1/2, c-Jun, JNK, and NF-κB.

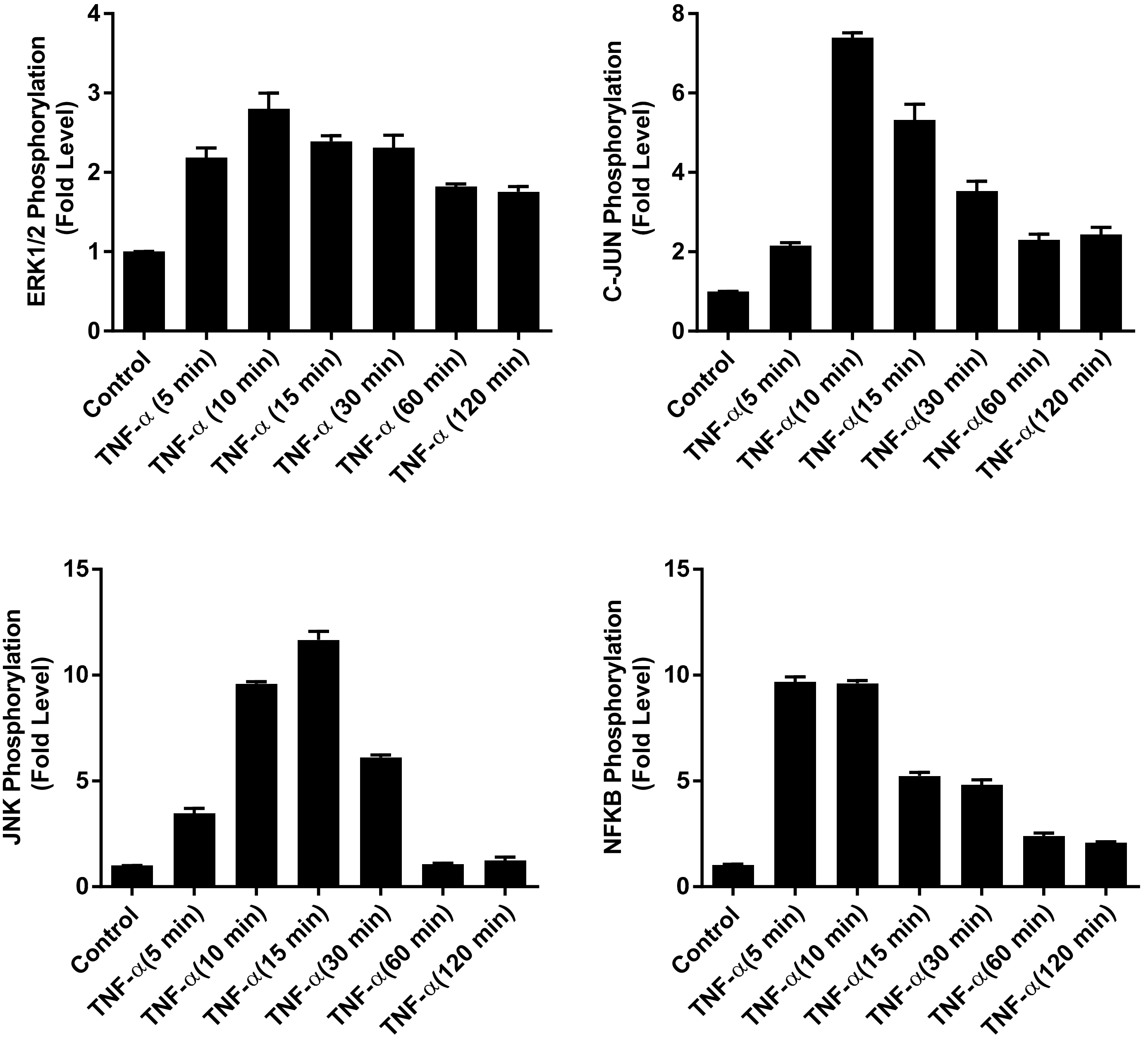

Figure S6. MAPK/NF-κB phosphorylation levels following TNF-α treatment

THP-1 monocytic cells were treated with TNF-α (10ng/mL) for 5, 10, 15, 30, 60, and 120 min. Cell lysates were centrifuged, and supernatants were collected for protein measurement. Samples (20µg each) were loaded and resolved by SDS-PAGE (12%) as described in materials and methods. Electroblotted proteins were developed to measure the expression of phosphorylated ERK1/2, c-Jun, JNK, and NF-κB

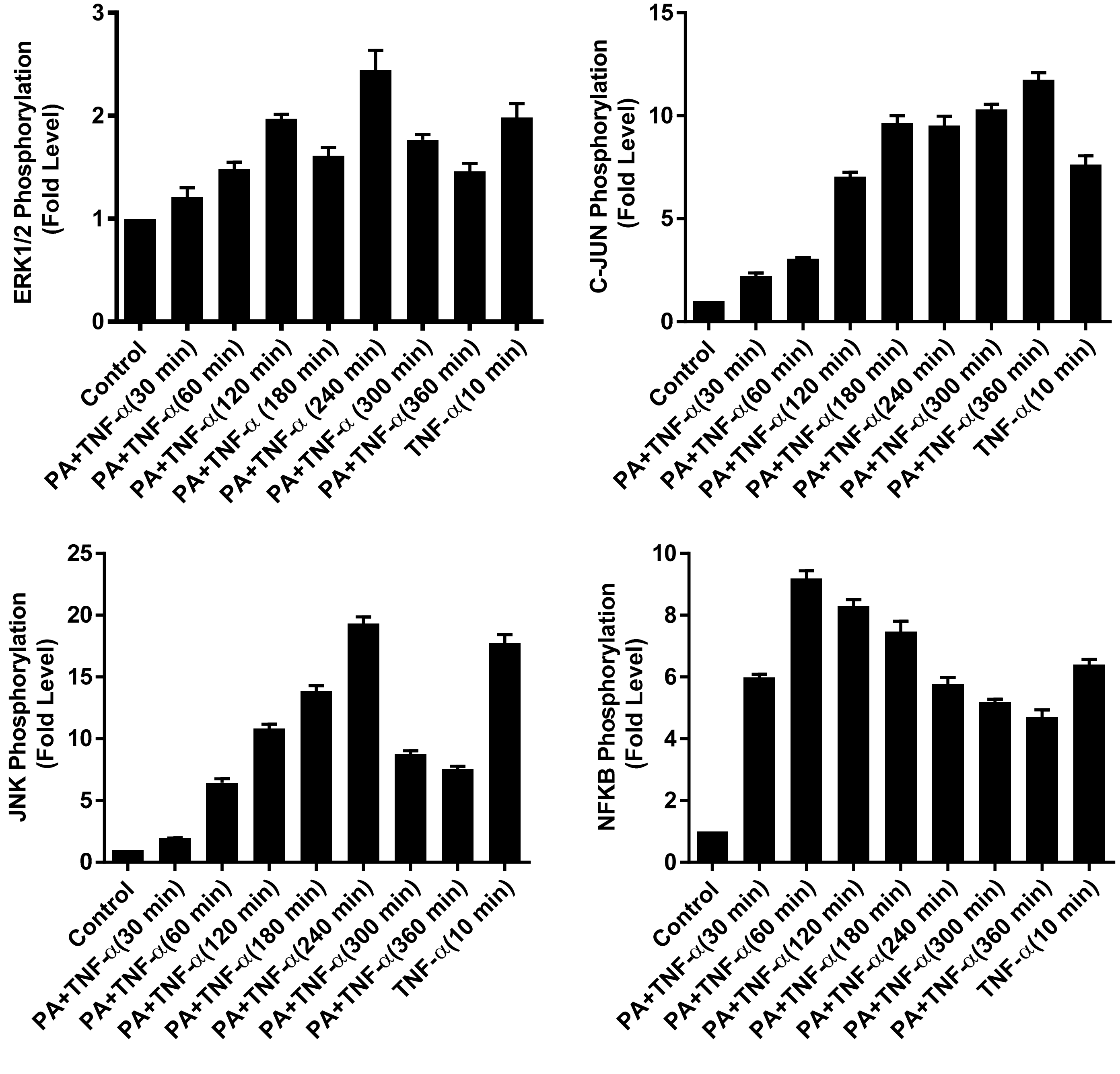

Figure S7. MAPK/NF-κB phosphorylation levels following palmitate/TNF-α co-treatment

THP-1 monocytic cells were co-treated with palmitate (200µM) and TNF-α (10ng/mL) for 5, 10, 15, 30, 60, and 120 min. Cell lysates were centrifuged, and supernatants were collected for protein measurement. Samples (20µg each) were loaded and resolved by SDS-PAGE (12%) as described in materials and methods. Electroblotted proteins were developed to measure the expression of phosphorylated ERK1/2, c-Jun, JNK, and NF-κB
